# Sphingomyelin synthase 2 promotes the stemness of breast cancer cells via modulating NF-κB signaling pathway

**DOI:** 10.1007/s00432-023-05589-y

**Published:** 2024-01-29

**Authors:** Haizhan Feng, Yahui Dong, Kunling Chen, Zicong You, Junyan Weng, Peiqiao Liang, Fujun Shi

**Affiliations:** 1 Department of Breast Surgery, Zhujiang Hospital, Southern Medical University, Guangzhou, China; 2 Department of Hepatobiliary Surgery II, Zhujiang Hospital, Southern Medical University, Guangzhou, China

**Keywords:** Sphingomyelin synthase 2, Cell stemness, Flotillin 2, NF-κB, Sphingomyelin, Drug resistance

## Abstract

**Objectives:**

Multi-drug resistance (MDR) to chemotherapy is the main obstacle influencing the anti-tumor effect in breast cancer, which might lead to the metastasis and recurrence of cancer. Until now, there are still no effective methods that can overcome MDR. In this study, we aimed to investigate the role of sphingomyelin synthase 2 (SMS2) in breast cancer resistance.

**Methods:**

Quantitative RT-PCR analysis was performed to assess changes in mRNA expression. Western blot analysis was performed to detect protein expression. Inhibitory concentration value of adriamycin (ADR) was evaluated using CCK 8 assay. The stemness ability of breast cancer cells was assessed by spheroid-formation assay. Immunofluorescence staining was conducted to show the cellular distribution of proteins. Breast tumor masses were harvested from the xenograft tumor mouse model.

**Results:**

SMS2 overexpression increased the IC50 values of breast cancer cells. SMS2 decreased the CD24 transcription level but increased the transcription levels of stemness-related genes including CD44, ALDH, OCT 4 and SOX2 in breast cancer cells. SMS2 overexpression promoted the nuclear translocation of phosphorylated NF-κB, while suppression of SMS2 could inhibit the NF-κB pathway.

**Conclusions:**

SMS2 increased the stemness of breast cancer cells via NF-κB signaling pathway, leading to resistance to the chemotherapeutic drug ADR. Thus, SMS2 might play a critical role in the development of breast cancer resistance, which is a previously unrecognized mechanism in breast cancer MDR development.

**Supplementary Information:**

The online version contains supplementary material available at 10.1007/s00432-023-05589-y.

## Introduction

Breast cancer is the most frequently diagnosed cancer in females worldwide (Ferlay et al. 2019). As a serious threat to women's physical and mental health, breast cancer is one of the main causes of cancer death in women. For patients with breast cancer with locally advanced or metastatic tumors, chemotherapy is the primary and standard treatment and adriamycin is applied as the first-line chemotherapy medication. However, most patients with breast cancer gradually develop resistance during chemotherapy. The efficacy of chemotherapy becomes gradually deteriorates as certain cancer cell populations become resistant to chemotherapy drugs, which results in the recurrence and progression of cancers. Until now, the molecular mechanisms of multidrug resistance (MDR) are very complicated and remain largely unclear. Therefore, a deeper understanding of the underlying mechanisms of chemoresistance will facilitate the development of new strategies for the treatment of patients with breast cancer.

MDR, defined as the resistance of cancer cells to chemotherapeutic drugs that may have different structures and mechanisms of action, is the major cause of failure in cancer chemotherapy. Previous studies indicated that multiple mechanisms participate in MDR, including increased drug efflux, drug inactivation, alteration in the drug target, decreased apoptosis, increased cancer stemness and increased DNA repair (Holohan et al. 2013). Until now, the molecular mechanisms of MDR are very complicated and have not been fully elucidated yet.

There is increasing evidence that a subpopulation multipotential differentiation, known as cancer stem cells (CSCs), promotes tumor formation, progress, metastasis and resistance to therapy. They lead to refractory cancer and poor prognosis (Ajani et al. 2015; Li et al. 2008). Antitumor drug resistance is an important property and characteristic of CSCs. Tumor cells can be reprogramed to dedifferentiate and obtain CSC properties by activating Notch, NF-κB, WNT/β-Catenin and JAK/STAT signaling pathways, which play central roles in stem cell maintenance (Eun et al. 2017).

At the plasma membrane, lipid rafts (LR) are specialized membrane microdomains enriched in cholesterol, sphingomyelin, and glycosphingolipids. As liquid-ordered structures with weak fluidity, LRs are involved in a variety of cellular physiological processes, such as cell signal transduction, material transportation, cell adhesion and migration, and play important roles in the development and progression of cancer (Eun et al. 2017; Mollinedo and Gajate 2015). Flotillins (also known as the Reggie family), the scaffolding proteins be reprogramed flotillin 1 and flotillin 2 (FLOT2). The gene encoding for FLOT2 is located on chromosome 17q11-12. FLOT2 is overexpressed and correlated with significantly worse prognosis in a variety of types of malignant tumors, including breast cancer, melanoma, stomach cancer, cervical cancer and nasopharyngeal cancer (Liu et al. 2015; Zhao et al. 2015). As an important regulator of oncogenic signaling, FLOT2 participates in the development of cancer via PI3K/AKT/MAPK, PI3K/AKT/mTOR, PI3K/AKT/FOXO and NF-κB signaling pathways.

Sphingomyelin synthase 2 (SMS2), located on the cell membrane, catalyzes the synthesis of sphingomyelin (SM) and diglyceride with ceramide and lecithin as substrates. SMS2 is a key enzyme to maintain the cellular homeostasis of ceramide and SM (Chen and Cao 2017). As an essential component of the lipid raft domain, SM provides the beneficial lipid microenvironment for signaling proteins on LR (Quinn 2014). Thus SMS2 plays an important role in processes involving SM and also participates in inflammation, atherosclerosis, proliferation, apoptosis and other biologic behaviour.

A recent study has proposed that SMS2 promotes the proliferation and migration of breast cancer cells (Zheng et al. 2019). In our study, we identified that SMS2 upregulated the expression of FLOT 2 and enhanced the stemness of breast cancer cells through NF-κB signaling pathway, which eventually resulted in increased resistance to the chemotherapeutic drug adriamycin (ADR). SMS2 may be a new strategy for the research of MDR in breast cancer.

## Material and methods

### Breast cancer cell lines

The breast cancer cell lines MCF-7 and MDA-MB-231 were obtained from the Cell Bank of the Chinese Academy of Science (Shanghai, China) and cultured in standard conditions as the protocol required. All cells were grown in RPMI-1640 medium (Life Technologies Corporation; Grand Island, NY) supplemented with 10% fetal bovine serum (Life Technologies Corporation; Grand Island, NY) at 37 °C with a humidity of 5% CO^2^.

### Quantitative RT-PCR analysis

Total RNA was extracted using Trizol (Invitrogen; Carlsbad, CA) and quantified by a NanoDrop ND1000 spectrophotometer (Thermo Fisher Scientific). The total RNA was subjected to polyadenylation and reverse transcription using a ThermoScriptTMRT-PCR System (Invitrogen). To quantify the mRNA levels of SMS2, FLOT2, CD44, CD24, OCT4, SOX2 and NANOG, Real-time PCR analysis was carried out using SYBR Green PCR master mix (Applied Biosystems; Foster City, CA) on an ABI 7500HT system. Data were normalized to GAPDH, and quantities were calculated using the 2 − ΔΔCt method.

### Western blot analysis

Protein expression was assessed by immunoblot analysis of cell lysates (20–60 μg) in RIPA buffer in the presence of rabbit antibodies against SMS2, FLOT2, CD24, CD44, OCT4, SOX2, NF-κB, IκB, Phospho-NF-κB, Phospho-IκB and mouse antibodies against IκB and GAPDH (1:1000; Cell Signaling Technology; Danvers, MA).

### IC50 and cell viability assay

Inhibitory concentration value (IC50) of ADR was evaluated using CCK 8 assay. MCF-7 and MDA-MB-231 cells were plated at a density of 1 × 10^4^ cells per well in 96-well plates and cultured at 37 °C with a humidity of 5% CO2 overnight. The cells were then treated with different concentrations of ADR for 24 h while glucose was added to the untreated cells as control groups.

### Sphere formation assay

The MCF-7 and MDA-MB-231 cells were plated at a density of 1000 cells per well in ultralow attachment plates (Corning, USA) with medium supplemented with 2% B-27, 20 ng/ml bFGF, and 20 ng/ml EGF. After 14 days, spheres < 50 μm in diameter were counted at 40 × magnification under a microscope.

### Nuclear and cytoplasmic fractionation

Cytoplasmic Extraction Reagent kit (#78833, Thermo Fisher Scientific, USA) was used to perform the nuclear and cytoplasmic fractionation according to the manufacturer's protocol.

### Immunofluorescent staining

Breast cancer cells MCF-7 and MDA-MB-231 (2.5 × 10^5^), cultured on glass coverslips, were fixed with 4% paraformaldehyde for 30 min after washing twice with PBS. Cells were then permeabilized with 0.1% Triton X-100 for 5 min and blocked with complete serum for 30 min. After incubating with primary antibodies of interest at 4 °C overnight, the cells were followed by an Incubation of Texas Red-conjugated anti-mouse or anti-rabbit antibody (Santa Cruz Biotech; USA) for 1 h. The cell nuclei were stained with DAPI for 10 min. Images were obtained under a laser scanning confocal imaging system (Olympus Microsystems).

### Xenograft tumor mouse model

Animal handling and experimental procedures were approved by the Institutional Animal Care and Use Committee of Southern Medical University (Guangzhou, China). Female nude BALB/c mice (4–6 weeks) were provided by the Laboratory Animal Services Centre at the Southern Medical University and maintained under sterile conditions during the experiment. 1 × 10^7^ SMS2 overexpression cells were injected subcutaneously into the right fat pad of nude mice, while the control cells were correspondingly injected into the left one. After 7 days, the mice were randomly assigned to one of two groups (*n* = 5/group). An equal concentration of ADR 1 mg/kg of body weight diluted in glucose (GS) was injected intraperitoneally to mice for 15 days at 4 days interval. The control mice were treated with GS. And tumor dimensions were measured every 4 days using a digital caliper (#03000002, GuangLu, China). Tumor volume was calculated according to the formula (Volume = 1/2 Length × Width^2^). On day 15, mice were sacrificed by cervical dislocation and breast tumor masses were quickly harvested for further experiments.

### Statistical analysis

Each assay was performed three times. Data were analyzed using SPSS statistic software version 19.0 (SPSS; Chicago, USA). All values are depicted as a mean ± standard deviation and statistical significance was established as *P* < 0.05. Student's *t* test and one-way ANOVA were carried out for comparisons between two groups. Paired-samples *t* test was used to analyze paired data. All statistical tests were two-sided.

## Results

### SMS2 promotes the ADR resistance of breast cancer cells

**Fig. 1:**
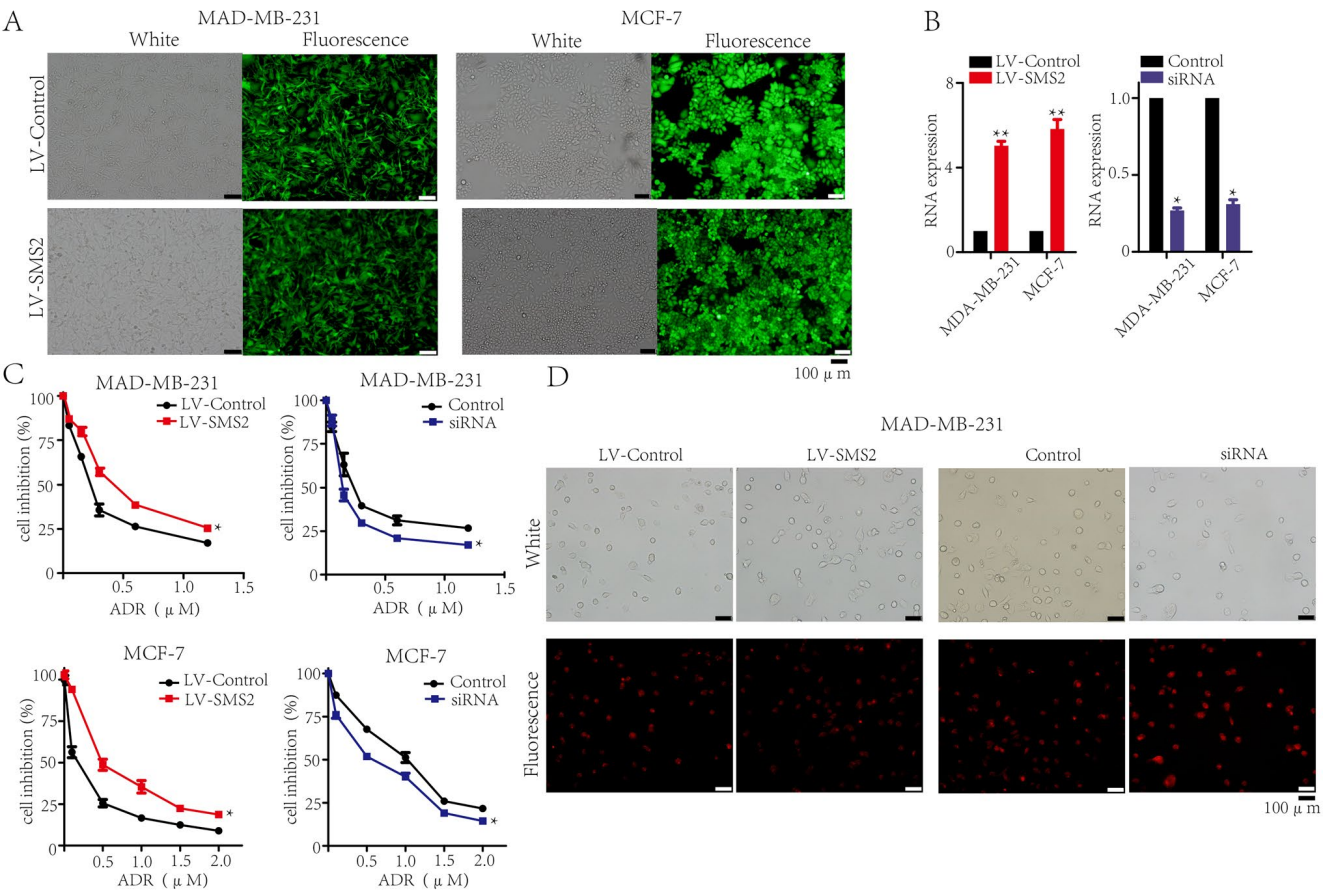
SMS2 promotes the ADR resistance of MCF-7 and MDA-MB-231 cells. **A** Immunofluorescence assay of SMS2 expression in breast cancer cells treated with SMS2 overexpressed-lentivirus or control-lentivirus. **B** Real-time PCR assays were performed to determine the efficiency of LV-SMS2 and SMS2 siRNA on MCF-7 and MDA-MB-231 cells. All samples were normalized against GAPDH. **C** The drug sensitivity to ADR was detected using CCK8 analysis. The results are from three independent experiments. *P < 0.05 and **P < 0.01 versus Control. **D** Immunofluorescence assay of intracellular concentration of ADR in MDA-MB-231 cells treated with SMS2 overexpressed-lentivirus, control-lentivirus or SMS2 siRNA

To investigate the effect of SMS2 on the chemotherapy resistance of breast cancer cells, we used two typical human breast cancer cell lines, MCF-7 and MDA-MB-231 cells. We successfully constructed the SMS2 overexpressed lentivirus (LV-SMS2) and synthesized the anti-SMS2 small interfering RNA (siRNA) (Table 1). Transfection efficiency of LV-SMS2 and siRNA in MCF-7 and MDA-MB-231 cells was confirmed by both fluorescence imaging (Fig. 1A), Real-time PCR (Fig. 1B) and western blot assays (Fig. 3C). The half-maximal inhibitory concentration (IC50) values of ADR were detected with CCK-8 assays in these cells. The results showed that SMS2 overexpression increased the IC50 values and the sensitivity to ADR subsequently decreased, whereas SMS2 interference elicited the opposite effect (*P* < 0.05, Fig. 1C). Fluorescence microscopy assays showed that SMS2 decreased the intracellular concentration of ADR in MDA-MB-231 cells (Fig. 1D), which was consistent with the above-described results. These findings suggest that SMS2 promotes the resistance to ADR of breast cancer cells in vitro.

**Table 1:** siRNA sequences for SMS2

Gene	Sense	Antisense
siRNA	CGAUUAGAAAGAUGAACAATT	UUGUUCAUCUUUCUAAUCGTT

### SMS2 promotes the cell stemness of breast cancer cells

**Fig. 2:**
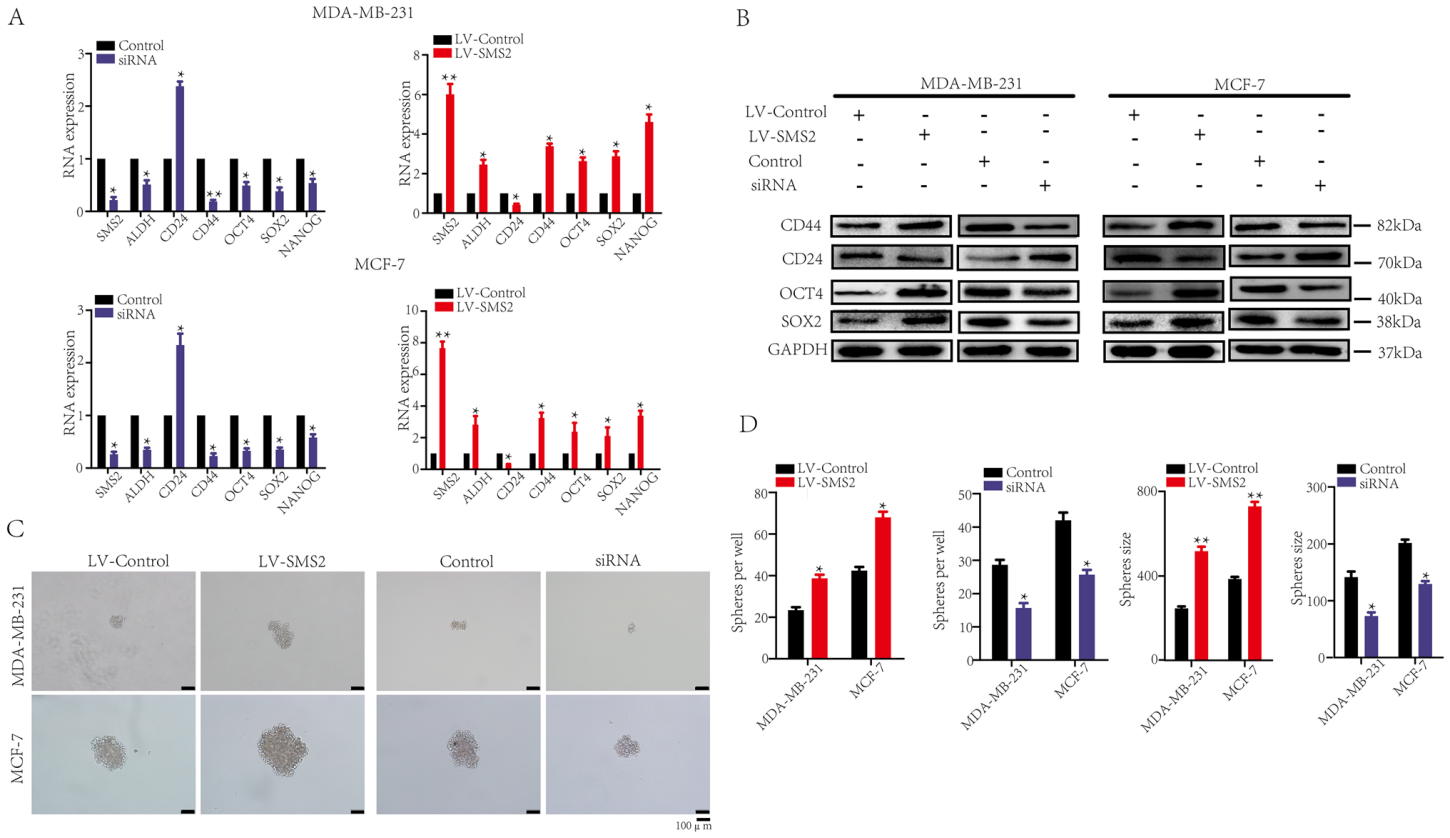
Effect of SMS2 on the stemness of breast cancer cells. **A** Transcript levels of stemness-related proteins in breast cancer cells treated as indicated were quantified by Real-time PCR and normalized against GAPDH transcript levels. **B** Western blot analysis was performed to examine the stemness-related proteins expression in breast cancer cells treated as indicated. GAPDH was used as inner control. **C** Images of tumor sphere formation assay detected the stemness of breast cancer cells. Scale bars represent 100 μm. **D** Sphere-forming analyses of number and size of the tumor spheres in breast cancer cells treated as indicated. The results are from three independent experiments. *P < 0.05 and **P < 0.01 versus Control

Emerging evidence showed that the CSC phenotype is one of the key characteristics of chemoresistance in tumor cells. CD24lo/CD44hi (expressing high levels of CD44 and low levels of CD24) is a major hallmark of breast cancer stem cells. Following this, we hypothesized that SMS2 enhanced the stemness of breast cancer cells and then promoted chemotherapy resistance to ADR. We further examined the SMS2 effects on the stemness-related genes and sphere formation in MCF-7 and MDA-MB-231 cells. Real-time PCR analysis showed that SMS2 decreased CD24 transcription level but increased the transcription levels of stemness-related genes including CD44, ALDH, OCT 4 and SOX2 in breast cancer cells (*P* < 0.05, Fig. 2A, Table 2). And we confirmed the Real-time PCR results with the western blot assays (Fig. 2B). Contrastingly, suppression of SMS2 yielded the opposite results. The stemness ability of breast cancer cells was assessed by spheroid-formation assay. In sphere-forming assays, overexpression of SMS2 in MCF-7 and MDA-MB-231 cells led to a significant increase in sphere formation ability in both the sphere's size and number (*P* < 0.05, Fig. 2C, D). Conversely, suppression of SMS2 decreased the sphere formation ability. We thus conclude that SMS2 can enhance the stemness characteristics of breast cancer cells, which may be the mechanism of SMS2 in chemoresistance promotion.

**Table 2:** RT-PCR primer sequences for human genes

Gene	Forward primer	Reverse primer
SMS2	CTTAGCCCTCCACTCCC	CAGAATCTGCGTCCCAC
FLOT2	TCGGAGGGGGTTCCACTATT	TTGGCTGCATCCCCGTATTT
CD44	TTACAGCCTCAGCAGAGCAC	TGACCTAAGACGGAGGGAGG
CD24	ATCTCCTCTTTGTGCCGGTT	CCGAAGCCCTTGCTTTGTTC
NANOG	TGGGAAGAAGCTAAAGAGCCAG	GGATGCTTCAAAGCAAGGCA
OCT4	TGACCGCATCTCCCCTCTAA	TCTTCCCAGAGGGAGCTCAA
SOX2	CATGAAGGAGCACCCGGATT	TTAATGTGCGCGTAACTGTG
GAPDH	GGAGCGAGATCCCTCCAAAAT	GGCTGTTGTCATACTTCTCATGG

### SMS2 upregulates the expression of lipid raft marker protein FLOT2 via SM

**Fig. 3:**
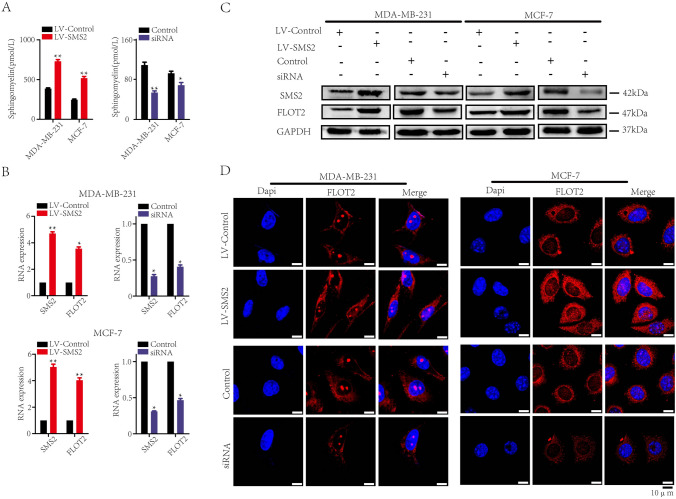
SMS2 upregulates the expression of lipid raft marker protein FLOT2 via SM. **A** ELISA assays of SM expression in MDA-MB-231 and MCF-7 cells treated as indicated. **B** Real-time PCR analysis was performed to test the transcript levels of intracellular SMS2 and FLOT2 in breast cancer cells treated as indicated. All samples were normalized against GAPDH. **C** Western blot analysis was performed to examine the intracellular SMS2 and FLOT2 expression in breast cancer cells treated as indicated. GAPDH was used as inner control. **D** Immunofluorescence assays of FLOT2 expression in treated cells as indicated. Representative figures are shown. Scale bars represent 10 μm. The results are from three independent experiments. *P < 0.05 and **P < 0.01 versus Control

SMS2 is the rate-limiting enzyme involved in the biosynthesis of SM. Enzyme-linked immunosorbent (ELISA) assays demonstrated that SMS2 significantly increased the expression of SM, while SMS2 suppression inhibited the expression of SM (*P* < 0.05, Fig. 3A). As a structural component of the cell membrane, especially of LRs, SM plays pivotal roles in preserving membrane fluidity and function and regulates various cellular processes (Ohnishi et al. 2017). We hypothesized that SMS2 may alter LRs functions by regulating membrane SM content. FLOT2, known as an LR marker protein, closely correlates with the stage and prognosis of breast cancer. Therefore, it is conceivable that SMS2 deficiency may have an impact on the expression of FLOT2. Real-time PCR suggested that SMS2 upregulated the transcriptional level of FLOT2 (*P* < 0.05, Fig. 3B). And Western blot assays also showed that SMS2 overexpression promoted the expression of FLOT2, while suppression of GMS2 presented the opposite result (Fig. 3C). Further immunofluorescence assays also determined that SMS2 upregulation stimulated expression of FLOT2, whereas inhibition of SMS2 expression suppressed the expression of FLOT2 (Fig. 3D).

### SMS2 enhances the ADR resistance of breast cancer in vivo

**Fig. 4:**
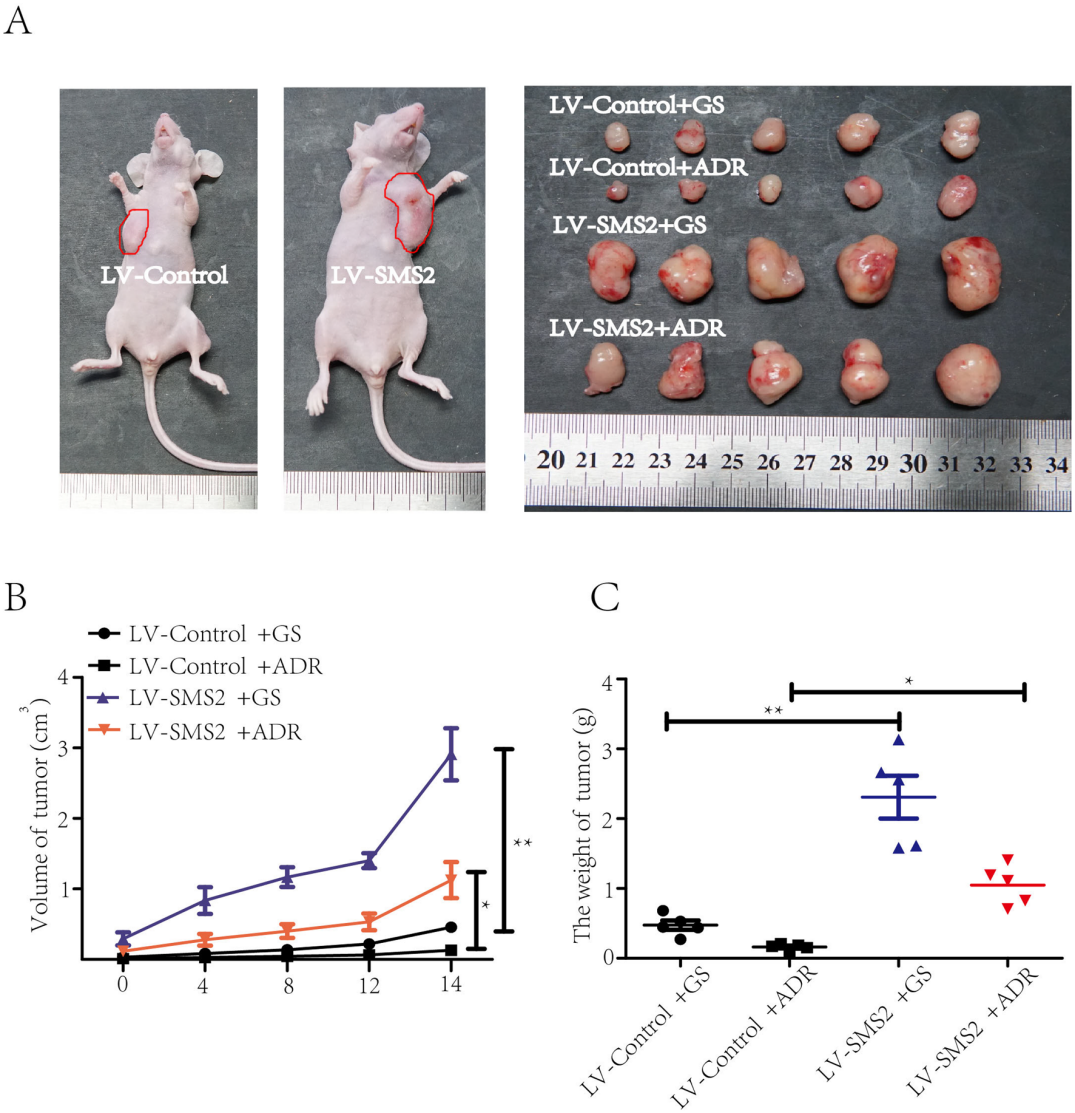
SMS2 overexpression promotes breast cancer ADR resistance in vivo. **A** Representative images of tumors formed in different treatment groups obtained after the 15 days therapy. Subcutaneous tumors were bigger in SMS2 overexpression groups than in control groups. The sizes of subcutaneous tumors were bigger in ADR groups than glucose (GS) groups. **B** Tumor growth curves of orthotopic tumors in the fat pad of nude mice after receiving ADR or glucose (GS) treatment. Tumor volumes were measured every 4 days. **C** Tumor volumes in nude mice 28 days after injections in different groups. *P < 0.05 and **P < 0.01 versus Control

To further investigate the impact of SMS2 on chemoresistance to ADR in vivo, MDA-MB-231 cells stably overexpressing SMS2 or control cells were injected into the mammary fat pads of 4-week-old female nude mice. We found that SMS2 significantly strengthened the resistance of MDA-MB-231 cells to ADR and reduced the chemotherapeutic effectiveness (Fig. 4A), which was consistent with the in vitro results. Compared with corresponding control groups, the nude mice that received SMS2-overexpressing MDA-MB-231 cells developed tumors more rapidly and grew bigger tumors after ADR treatment (*P* < 0.05, Fig. 4B, C). In addition, we also found that SMS2 overexpression significantly promoted tumor growth in vivo, shown in the nude mice without ADR treatment (*P* < 0.05, Fig. 4B, C).

### SMS2 enhances the stemness of breast cancer cells by activating the NF-κB signaling pathway

**Fig. 5:**
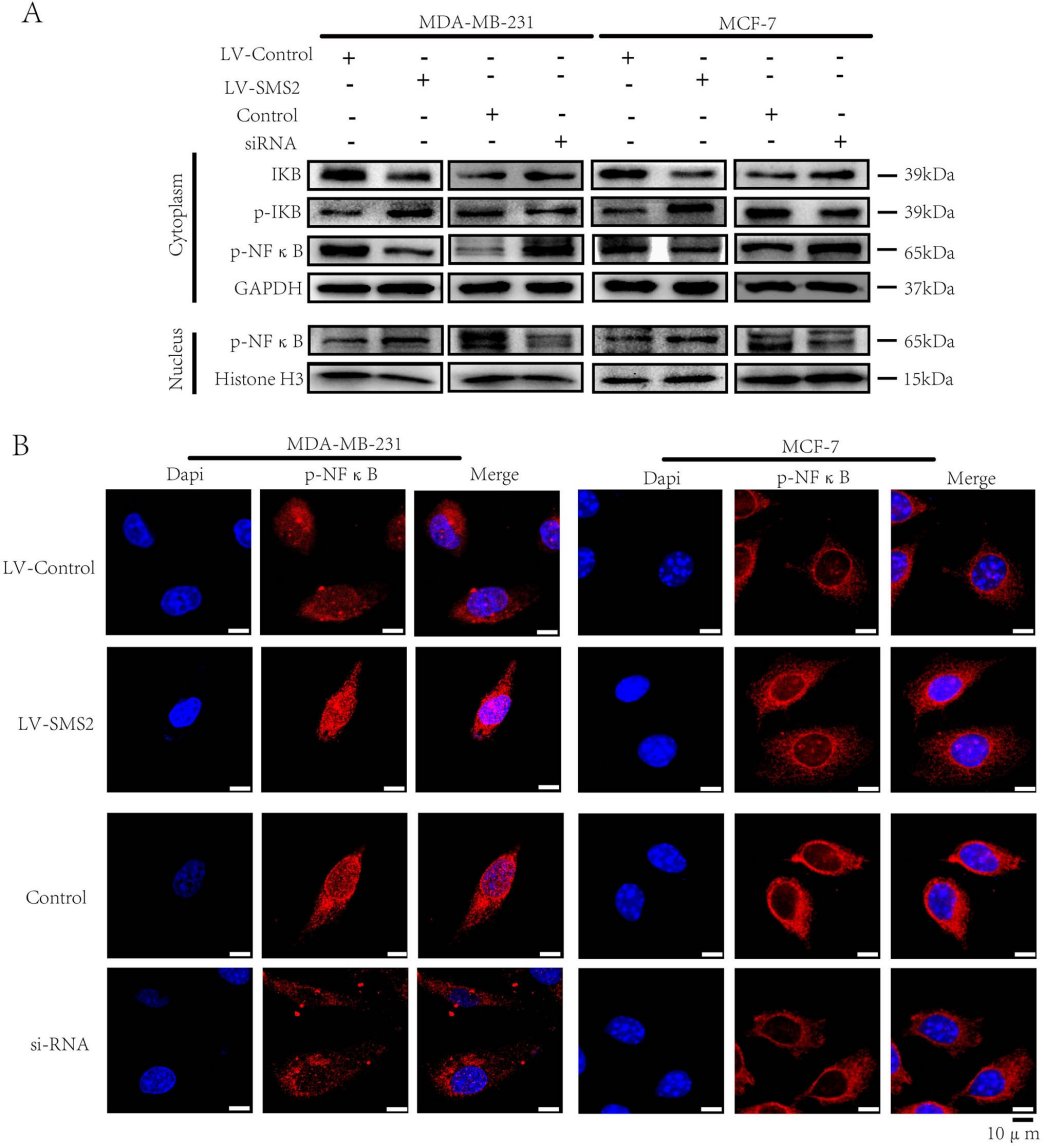
SMS2 enhances the stemness of breast cancer cells by activating the NF-κB signaling pathway. **A** Western blot analysis was performed to detect NF-κB signaling pathway-associated proteins. GAPDH was used as a control in the cytoplasm and Histone H3 was used as a control in the nucleus. **B** Immunofluorescence assays of p-NFκB expression in treated cells as indicated. Representative figures are shown. Scale bars represent 10 μm. *P < 0.05 and **P < 0.01 versus Control

NF-κB signaling has been documented to be a key regulator of cancer stemness. It is reported that FLOT2 could activate the NF-κB signaling to promote cancer development. To define the mechanism by which SMS2 enhances the stemness of breast cancer cells, we investigated the NF-κB signaling pathway. The entry of NF-κB from the cytoplasm to the nucleus after phosphorylation is an important marker for activation of NF-κB signaling. Western blot assay indicated a stimulatory role of SMS2 on the nuclear translocation of NF-κB. Meanwhile, the expression of inhibitor NF-κB alpha (IκBα) was relatively attenuated (Fig. 5A). Consistently, immunofluorescence staining showed that SMS2 overexpression promoted the nuclear translocation of phosphorylated NF-κB (p-NFκB), while suppression of SMS2 could inhibit the NF-κB pathway (Fig. 5B).

## Discussion

In mammals, there are three isoforms for SMSs, including SMS1, SMS2 and SMSr, while only SMS1 and SMS2 are responsible for SM synthesis. SMS2 primarily localizes at the plasma membrane, whereas SMS1 primarily resides in the Golgi apparatus (Chen and Cao 2017). SMS2 works as the last enzyme involved in the biosynthesis of SM and generates SM from ceramide and phosphatidylcholine. SM plays a critical role in the constitution of membrane microdomains such as LR and caveolae, which implicates the regulation of trans-membrane signaling. Recent studies have shown the crucial roles of SMS2 in the regulation of transmembrane signaling related to cell proliferation or inflammation by modulating the SM/ceramide balance (Ohnishi et al. 2017; Taniguchi et al. 2020). We previously indicated that the level of SMS2 protein expression was significantly high for metastasis-positive cases of breast cancer. And SMS2 promoted cell migration and invasion via EMT by regulating the TGF-β/Smad signaling pathway (Zheng et al. 2019). Overexpression of SMS2 promotes cancer progression and is associated with poor prognosis of breast cancer. However, its role and molecular mechanisms have not been fully clarified and need to be further elucidated.

The current studies have shown that lipometabolism has a great effect on the occurrence and development process of tumors. Many cancer cells present an enhanced lipid metabolism. And aberrant regulation of the sphingolipid metabolism is involved in the progression of malignancy and drug resistance (Tirinato et al. 2017). The lipid composition of cell membranes in drug-resistant cancer cells is slightly different from that of chemosensitive cancer cells. The former has higher SM contents which increase the membrane rigidity and hence decrease the fluidity of the cell membrane (Lafont et al. 2011). The regulation of SMS2 activity or expression can significantly alter the SM content of cell membrane, especially in LRs (Li et al. 2007). The inhibition of SMS activity is likely to alter membrane composition and properties through SM depletion in the LR and to regulate the function of trans-membrane receptors.

Our results also suggested that overexpression of SMS2 increased the content of SM in breast cancer cells. This alteration significantly enhanced the resistance of breast cancer cells toward ADR both in vitro and vivo, while the intracellular accumulation of ADR was decreased. SMS2 downregulation lessened the content of SM and sensitized the cells to ADR treatment. It strongly suggested that SMS2 was associated with ADR drug resistance in breast cancer. It was reported that the expression and function of P-glycoprotein were significantly downregulated in the SMS2-deficient brain (Zhang et al. 2011). A growing body of evidence indicated that SMSs modulated hematological cell proliferation and sensitivity to anticancer reagent-induced apoptosis (Taniguchi and Okazaki 2014). But our further study found the stimulative effect of SMS2 on cell stemness and it may be one of the major mechanisms by which SMS2 mediated drug resistance.

After the successful isolation of breast cancer stem cells (BCSCs) from cancer specimens by Al et al. in 2003, BCSCs have been considered essential contributors to metastasis and therapeutic resistance in BC patients (Al-Hajj et al. 2003; Mehraj et al. 2021). Many studies have shown that cancer stem cells (CSCs) are more resistant to conventional therapies in TNBC, which cause survival and relapse of cancer (Merikhian et al. 2021). Recent studies have shown that CSCs are another important factor in the failure of chemotherapy (Kim et al. 2017; Leon et al. 2016). Several lines of evidence indicate CSCs enrichment following exposure to chemo drugs, resulting in multidrug resistance (MDR). As chemotherapy exclusively targets the proliferating fraction of tumor cells, the BCSCs can evade systemic therapies, and in turn, develop drug resistance (Saha and Lukong 2022). CSCs characterised by the expression of specific stem cell markers possess features of self-renewal, high tumor-initiating potential, differentiation potential and drug resistance (Lytle et al. 2018; Phi et al. 2018). And factors contributing to BCSC drug resistance against conventional therapeutic drugs include vasculogenic mimicry, decreased ferroptosis, increased autophagy, enhanced drug efflux, enhanced DNA repair and immune escape (Saha and Lukong 2022). The fully differentiated cancer cells can be reprogrammed into stem cell-like cells in various tumor types (Eun et al. 2017). It has been widely accepted that CD44hi/CD24lo breast cancer cells act as the stem cell-like population (Al-Hajj et al. 2003). Cancer cells with CSC-like phenotypes are more resistant to anti-cancer drugs (Song et al. 2017) and reducing cancer stemness can be an effective method to abrogate cancer chemoresistance. Targeting CSCs enhances triple-negative breast cancer responsiveness to chemotherapy (He et al. 2019). Our study revealed that SMS2 could enhance the stemness phenotypes of breast cancer cells and induce resistance to ADR. This could be related to the regulation of SM content in cell membrane by SMS2.

As one of the major lipids comprising the plasma membrane, SM constitutes membrane microdomains such as LR and implicates in the regulation of trans-membrane signaling. LRs, where SM is enriched and held together mainly by hydrophobic interactions, form platforms for the regulation and transduction of receptor-mediated signaling in cancer (Lingwood and Simons 2010; Mollinedo and Gajate 2015). Increasing evidence suggests that the inhibition of SMS2 leads to the SM depletion of cell membrane and it subsequently results in a disruption of SM-enriched microdomains and a regulation of lipid-raft-associated proteins (Taniguchi and Okazaki 2014). It was reported that the membrane SM was important for Fas clustering through aggregation of LRs, leading to Fas-mediated apoptosis (Miyaji et al. 2005). In apoptosis-resistant S49 mouse lymphoma cells, SM content was lower than the chemo-sensitive cells because of the inhibition of SMS, suggesting that maintenance of SM via SMS in the membrane was more critical to recover Fas-sensitivity than increase of ceramide amount (van Blitterswijk et al. 2009). SMS2 deficiency-mediated reduction of SM in lipid microdomains where insulin receptors located at improved insulin sensitivity, which was responsible for the improvement of obesity and type 2 diabetes (Li et al. 2011; Mitsutake et al. 2011). Our findings demonstrated that SMS2 upregulated the lipid raft marker protein FLOT2 in breast cancer cells. Conversely, the suppression of SMS2 interfered with FLOT2 expression. It may relate to the regulation of LR via conformational changes in lipids on the membrane by SMS2.

FLOT2 overexpression is reported to be associated with the tumor grade, TNM stage, distant metastasis in multiple malignancies and can be useful as a prognostic biomarker for breast cancer progression (Liu et al. 2018; Wang et al. 2013). As a regulator of lung metastasis, decreased expression of FLOT2 protein reduces the tumorigenic and metastatic ability of human breast cancer cell lines in vivo (Berger et al. 2013). It has been reported that FLOT2 participates in the development of several types of malignant tumors, which activates the PI3K/AKT/mTOR, PI3K/AKT/MAPK pathways and NF-κB signaling to promote cell proliferation, migration and invasion (Asp et al. 2014; Gong et al. 2013). The canonical NF-κB signaling pathway is well-known to serve an important role in stemness maintenance and regulation of diverse malignancies (Rinkenbaugh and Baldwin 2016). Non-CSC tumor cells can dedifferentiate and change their phenotype to a CSC-like phenotype via NF-κB signaling pathway. In addition, the inhibition of NF-κB signaling pathway reduced the expression of the CSC marker (Park et al. 2015) and suppressed breast CSC formation (Choi et al. 2017). To determine the mechanism underlying the improvement of stemness mediated by SMS2, we investigated the expression levels of the key proteins of NF-κB signaling pathway and found that SMS2 indeed activated the NF-κB signaling pathway via FLOT2. Recent studies have proposed that the decrease of the membrane SM/ceramide balance by SMS2 deficiency suppressed NF-κB activation (Prymas et al. 2020).

In our study, we found that SGMS can enhance the cell stemness of MDA-MB-231 and MCF-7 cells by activating the NF-κB signaling pathway, which is related to its improvement (regulation) of SM content and further promotion of FLOT2 expression. It is the Enhancement of cell stemness that prevents the chemotherapy cytotoxicity of ADR. However, it is reported that the knockdown of FLOT2 suppresses chemoresistance of HCT-15/ADM cells and the mechanism of this function may be associated with the activation of the PI3K/Akt signaling pathway (Ye et al. 2019). The underlying molecular mechanism via which SMS2 influences stemness and drug resistance in breast cancer needs to be further clarified. Our study revealed that SMS2-mediated activation of the NF-κB signaling pathway is important in breast cancer drug resistance. Targeting SMS2 may be a safe and efficacious new approach for breast cancer therapy.

Collectively, our findings indicated that the aberrant expression of SMS2 disrupted the homeostasis of SM, which was one of the major lipids comprising the lipid raft. SMS2 increased the expression of lipid-raft-associated FLOT2 by upregulating SM, which subsequently activated the NF-κB signaling pathway and promoted cell stemness of breast cancer cells, thus increasing the drug resistance toward ADR.

It is suggested that SMS2 makes an important contribution to the cell membrane structure and function. These findings may provide new insight into the mechanisms underlying breast cancer chemoresistance. In the future, novel therapeutic procedures for breast cancer could be developed through modifying SMS2.

### Supplementary Information

Below is the link to the electronic supplementary material.

Supplementary file1 (DOCX 16 KB)

## Data Availability

The datasets generated during and analyzed during the current study are available from the corresponding author upon reasonable request.

## Abbreviations

MDR: Multi-drug resistance
SMS2: Sphingomyelin synthase 2
SM: Sphingomyelin
FLOT2: Flotillin 2
ADR: Adriamycin
LR: Lipid raft
CSCs: Cancer stem cells
BCSCs: Breast cancer stem cells
