# Supplementary material for: Sphingomyelin synthase 2 promotes the stemness of breast cancer cells via modulating NF-κB signaling pathway
Source: J Cancer Res Clin Oncol. 2024 Jan 29;150(2):46. doi: 10.1007/s00432-023-05589-y (PMC10824874; doi:10.1007/s00432-023-05589-y)
Supplement: Supplementary file 1 — Supplementary file1 (DOCX 16 KB) [file 432_2023_5589_MOESM1_ESM.docx]

**Supplementary TableS1.** RT-PCR primer sequences for human genes

| **Gene** | **Forwardprimer** | **Reverseprimer** |
| --- | --- | --- |
| **SMS2** | **CTTAGCCCTCCACTCCC** | **CAGAATCTGCGTCCCAC** |
| **FLOT2** | **TCGGAGGGGGTTCCACTATT** | **TTGGCTGCATCCCCGTATTT** |
| **CD44** | **TTACAGCCTCAGCAGAGCAC** | **TGACCTAAGACGGAGGGAGG** |
| **CD24** | **ATCTCCTCTTTGTGCCGGTT** | **CCGAAGCCCTTGCTTTGTTC** |
| **NANOG** | **TGGGAAGAAGCTAAAGAGCCAG** | **GGATGCTTCAAAGCAAGGCA** |
| **OCT4** | **TGACCGCATCTCCCCTCTAA** | **TCTTCCCAGAGGGAGCTCAA** |
| **SOX2** | **CATGAAGGAGCACCCGGATT** | **TTAATGTGCGCGTAACTGTG** |
| **GAPDH** | **GGAGCGAGATCCCTCCAAAAT** | **GGCTGTTGTCATACTTCTCATGG** |

**Supplementary TableS2.** siRNA sequences for SMS2

| **Gene** | **Sense** | **Antisense** |
| --- | --- | --- |
| **siRNA** | **CGAUUAGAAAGAUGAACAATT** | **UUGUUCAUCUUUCUAAUCGTT** |
